# Practice of standard monitoring during anaesthesia in hospitals of North Kivu: a survey of health facilities of the health antenna of Butembo

**DOI:** 10.1186/s12913-020-05076-x

**Published:** 2020-03-30

**Authors:** Furaha Nzanzu Blaise Pascal, Paulin Kambale Musavuli, Joel Kambale Ketha, Franck Katembo Sikakulya, Andreas Barratt-Due, Thomas Castner, Gregor Pollach

**Affiliations:** 1 Department of Anaesthesia and Intensive care, College of Medicine, University of Malawi, Blantyre, Malawi; 2 Faculty of Medicine, Université Catholique du Graben, Butembo, Democratic Republic of the Congo; 3 Sainte Famille Hospital Center of Butembo, DRC, Butembo, Democratic Republic of the Congo; 4 Department of Anaesthesia and Critical Care Medicine, College of Medicine, University of Rwanda, Kigali, Republic of Rwanda; 5 Department of Surgery, Kampala International University, Kampala, Uganda; 6 Division of Emergencies and Critical Care, Rikshospitalet, Oslo University Hospital, Oslo, Norway

## Abstract

**Background:**

Standard monitoring during anaesthesia is a core element of patient safety and practice of safe anesthesia has reduced morbidity and mortality worldwide. The main objective of this study was to assess the practice of standard monitoring during anaesthesia in the hospitals of North Kivu, so as to establish a baseline overview of the situation, and orientate plans towards safe anaesthesia in the region.

**Methods:**

This study was a cross-sectional survey of health facilities of the Health Antenna of Butembo in Democratic Republic of Congo and was conducted from October to December 2018. Questionnaires were brought to anaesthesia providers in the health facilities. The study included 1 answer from the anaesthesia practitioners who accepted to participate. The practices of standard monitoring in the health facilities were compared to WHO-WSFA guidelines. Data was captured and analyzed with Epi Info 7.

**Results:**

Forty out of 90 health facilities (44.4%) of 10 health zones responded on the questionnaire. Twenty-three health facilities (57.5%) were from private sector and 17 (42.5%) from public sector. Sixteen health facilities (40.0%) were from the Butembo health zone. The median number of anaesthesia providers was 2 per health facility. Of all the anaesthesia providers, none were physicians, 47.5% were nurses practicing anaesthesia without any training in anaesthesia and 47.5% were nurse anaesthetists. All the health facilities were providing general anaesthesia whereas spinal anaesthesia was provided in 22 out of 40 centers (55%). Seventy percent (28/40) of the facilities were below standard according to WHO-WSFA guidelines. Only 40% (16/40) were using a pulse oximeter and 10% (4/40) declared that ECG was occasionally used.

**Conclusion:**

The practice of standard monitoring is poor in health facilities of the Health Antenna of Butembo. Efforts should be made to improve monitoring which is a key element of safe anaesthesia.

## Background

Access to safe anaesthesia is nowadays considered as an integral part of universal health coverage and a basic human right [1, 2]. Practice of safe anaesthesia has helped to reduce morbidity and mortality related to anaesthesia over the world [2–4]. Recently, Pignaton et al. reported that anesthesia-related mortality had been reduced from 1.12 per 10,000 to zero in Brazil [5]. However, in low-income countries, the rates are still very high. Greater risks of anesthesia-related mortality, between 1:133 and 1:1925, have been reported [3, 4, 6].

Improvement in monitoring has been recognized as a contributing factor towards such achievement together with suitable, available and well maintained equipment, infrastructure and increasing number of well-trained anaesthesia providers worldwide [1, 3, 4, 7]. In fact, the use of standard monitors is a core element for patient safety. Standard monitoring includes the clinical observation by an appropriately trained anaesthesia provider and continuous evaluation with appropriate monitors of the patient’s oxygenation, ventilation, circulation and temperature [1, 2].

The world health organization (WHO) and the world federation of societies of anaesthesiologists (WFSA) have recently and jointly published International standards for safe practice of anaesthesia. They have classified monitoring standards according to highly recommended, recommended and suggested, and considered anaesthesia unsafe and unacceptable if highly recommended standards are lacking [1, 8]. The highly recommended standards include the continuous presence of a trained and vigilant anaesthesia provider, continuous monitoring of tissue oxygenation and perfusion by clinical observation and a pulse oximeter, intermittent monitoring of blood pressure, confirmation of correct placement of an endotracheal tube by auscultation and carbon dioxide detection. Additionally, the use of the WHO Safe Surgery Checklist and a system for transfer of care at the end of anaesthesia are highly recommended [1].

However, in low-income countries, the constraints related to poor resources make it challenging to achieve appropriate monitoring standards. Patients are still anaesthetized most likely in unsafe conditions [3, 4]. The aim of this study was to assess the practice of standard monitoring during anaesthesia in the hospitals of North Kivu in order to have a baseline overview of the situation, which may help in orienting plans for actions towards safe anaesthesia in the region.

## Methods

**Fig. 1:**
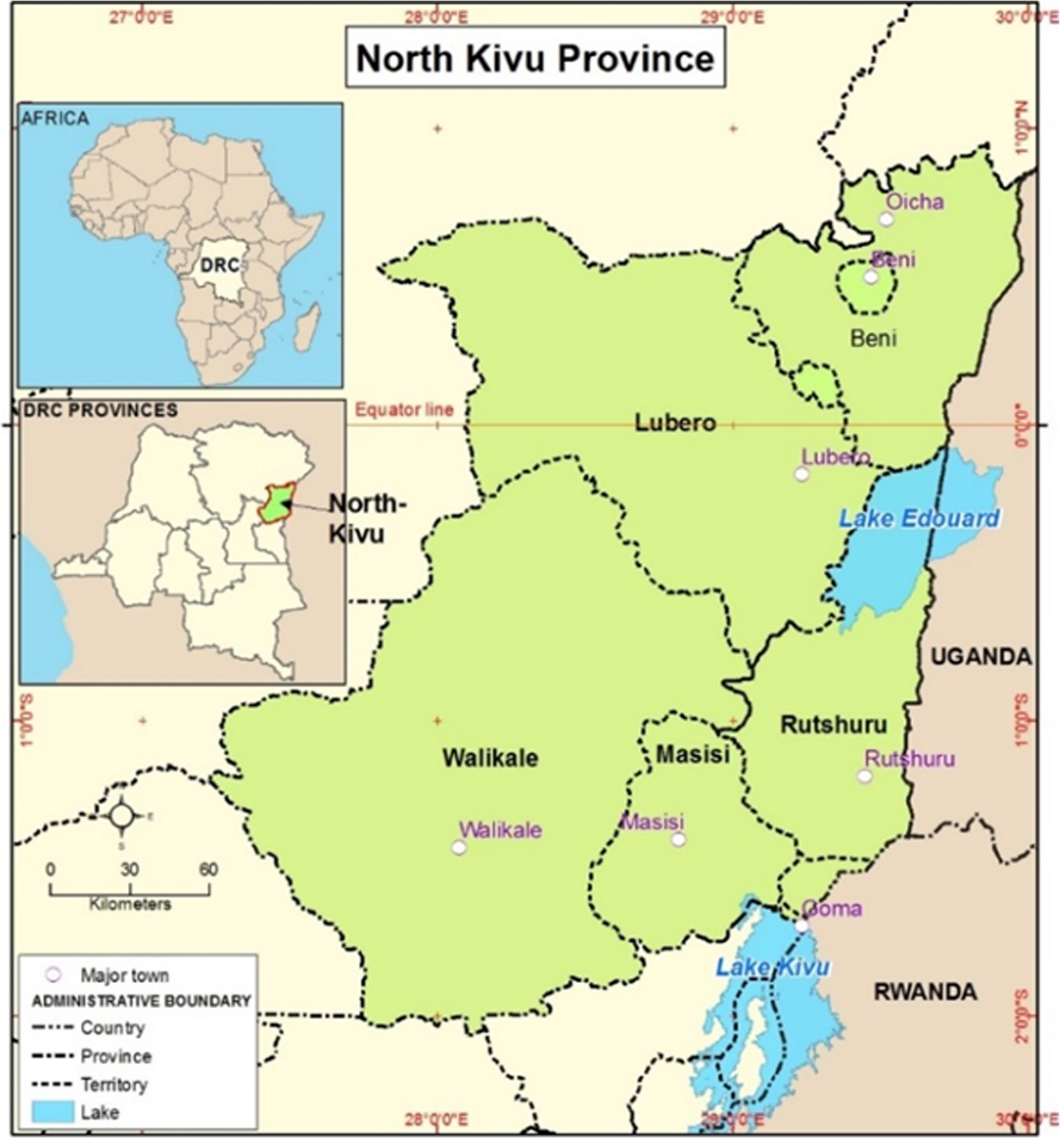
The North Kivu Province showing the Beni and Lubero territories [9]. (Source: http://article.sapub.org/10.5923.j.ajgis.20190802.01.html)

This study was conducted in referral hospitals and surgical centers of the Health Antenna of Butembo. The Health “Antenna” of Butembo, which is a subdivision of the North Kivu Health Division, covers the Lubero and Beni territories as well as the Beni and Butembo cities in the North Region of the North Kivu Province in the Eastern part of Democratic Republic of Congo (DRC) (Fig. 1). The Health Antenna heads 17 Health Zones. In total, surgeries are performed in 90 health facilities. Seventeen are General Referral Hospitals, where normally major operations are happening, and 63 Health facilities have small surgery units. The number of beds in the referral hospitals varies from 100 to 300 beds. The Health Antenna was chosen because it is representative of the situation in North Kivu facing different challenges; insecurity caused by the presence of armed groups, a dense population covering approximately 3.5 million inhabitants. Additionally, the region is facing Ebola outbreak since August 2018. The Anaesthesia faculty, in the local Nursing College, the “Institut Superieur des techniques médicales” (ISTM) of Butembo, offers an opportunity to access trained anaesthetic nurses in this zone. There is no training of doctors in Anaesthesia in the local universities.

This study was a cross sectional survey conducted from October 2018 to December 2018. The study population consisted of all anaesthesia providers of the departments or units of anaesthesia of the Health facilities in the Health Antenna of Butembo.

The study was based on convenience sampling, including anaesthesia providers in departments/units of anaesthesia in the study site who wanted to participate and whose departments/units were accessible geographically to the investigators with considerations to their safety and security. The anaesthesia providers who did not respond on the questionnaire or did not want to participate were not included.

Data were collected using a data collection form specifically designed for the survey of the anaesthesia providers in the department or unit (Additional fine 1). Questionnaires were brought in person to each anaesthesia department or unit of the health facility by investigators. The questionnaire was presented and explained to the members of the department or unit who were then requested to answer and handled it back within a month. The investigators collected the filled forms at deadline. Only one questionnaire for each health facility was included in the final analysis. When more than one responses were obtained from a facility, the answer of the most experienced in term of years of service and/or qualification was considered in the analysis.

The variables of the study included the identification of the facility, number of operating rooms, type of anaesthesia frequently practiced, number of anaesthesia providers, their qualification and level of education, number of anaesthesia providers present during anaesthesia and the age of the responding anaesthesia provider. The type of anaesthesia was either general anaesthesia or spinal anaesthesia. An anaesthesia provider was defined as any personnel giving anaesthesia during an operation regardless of his medical qualification. The term “Gradué” for the level of studies means that the anaesthesia provider did 3 more years of anaesthesia in the ISTM after a first qualification in nursing. Anaesthesia is a mature entry program requiring a degree in nursing to get admission into the program, thus the “Gradué” holds a degree in anaesthesia and is a qualified nurse anaesthetist. The “Licencié” is a qualified nurse anaesthetist who has done 2 more years of anaesthesia after the “Gradué” degree which makes a total of 5 years of anaesthesia training post nursing school. A “Licence” in Congolese system is an equivalent to Master in English LMD (License Master Doctorate) system. The health facilities were classified into 2 groups. The private sector included health facilities from purely private sector, not for profit and faith based organizations. The public sector included all facilities led directly by the government.

The other group of variables was the type of monitors used. This could either be a multi-parameter monitor or separate monitors measuring one parameter each. When a separate instrument monitored a single parameter, the type of blood pressure (BP) machine was determined as either a manual BP machine or an electronic one. The frequency of using each parameter of monitoring was classified into 3 groups; never, sometimes, and always. The reasons for not using monitors were recorded.

The standard monitoring in this study was defined as all the highly recommended standards for monitoring according to the joint definition of WHO and WFSA for intra-operative and postoperative monitoring. This includes the presence of a trained anaesthesia provider for clinical monitoring, the continuous use of pulse oximeter, the intermittent non-invasive blood pressure monitoring, and the audible signal and alarms at all time during anaesthesia (i.e. the use of electronic monitor) [1]. The health facilities were classified into 2 groups according to their monitoring practices compared to WHO and WFSA definitions. Group one included all health facilities that met the standard monitoring definition and was termed “Acceptable Standard Practice”, whereas Group two included the health facilities that did not meet the definition and was termed “Below standard Practice”.

### Data management and presentation

Data were captured and analyzed with Epi Info 7. Descriptive statistics were present in contingency tables with frequencies.

### Ethics

The study was authorized by the Academic Board of the Catholic University of Graben and approved by the University Ethics committee, the “Comité Ethique du Nord-Kivu”, under trial No 08/TEN/2018. The Health Antenna of Butembo Ethical Committee also approved the study. A written, informed and explained consent was obtained from all the participants before the survey and all received information has been anonymized. The study has been conducted according to good ethical practice.

## Results

### Health facilities and anaesthesia providers

We received feedback from 40 out of 90 health facilities (44.4%) of 10 health zones. Forty answers from these facilities constituted the sample for this study. Twenty-three health facilities (57.5%) were from private sector and 17 (42.5%) from public sector. Sixteen health facilities (40.0%) were from the Butembo health zone. The minimum number of operating rooms was 1 per health facility with a maximum of 3. Only 3 health facilities had 3 operating rooms. Table 1 gives the distribution of participants according to health zones and number of operating rooms.

**Table 1:** Distribution of participants according to health zones and number of operating rooms

Variables	Frequency (***N*** = 40)	Percentage
**Health Zone**		
Butembo	16	40.0
Katwa	15	37.5
Beni	2	5.0
Mabalako	2	5.0
Kalunguta	1	2.5
Mangurejipa	1	2.5
Masereka	1	2.5
Lubero	1	2.5
Vuhovi	1	2.5
**Number of Operating Room**		
1	33	82.5
2	4	10.0
3	3	7.5

The median number of anaesthesia providers was 2 per health facility with a minimum of 1 and the maximum of 5. The mean age of the interviewed anaesthesia providers was 34 years with a standard deviation (SD) of 9 years (min 21 years, max 57 years). No physicians were among the anaesthesia providers, 47.5% were nurses practicing anaesthesia without any previous training in anaesthesia and 47.5% were nurses with a “Gradué” degree in anaesthesia. The median duration of experience was 3.5 years with a minimum of 1 year and a maximum of 26 years. Table 2 gives the characteristics of anaesthesia providers.

**Table 2:** Characteristics of Anaesthesia providers

Variables	Frequency (***N*** = 40)	Percentage
**Number of Anesthesia providers per facility**		
1 to 2	27	67.5
3 to 4	12	30.0
5	1	2.5
**Level of anaesthesia training**		
Nurse without Anaesthesia training	19	47.5
Nurse with a degree in Anaesthesia	19	47.5
Nurse with a “Licence” in Anaesthesia	2	5.0
**Experience in years**		
1 to 3	22	55.0
4 to 6	7	17.5
7 to 10	8	20.0
More than 10	3	7.5
**Number of Anaesthesia providers during a case**		
Always one	23	57.5
Nurse under Doctor/Surgeon responsibility	11	27.5
Sometimes two	3	7.5
One with help of a nurse	2	5.0
Always 2	1	2.5

### Practices of standard monitoring

All the health facilities were practicing general anaesthesia. Spinal anaesthesia was practiced in 22 centers of the 40 (55%). A stethoscope was available in all the facilities. From the 40 anaesthesia providers, 24 (60%) were using a separate monitoring instrument for each monitoring parameter and all of them were using a manual Blood Pressure (BP) machine to monitor the BP. The remaining 16 (40%) were using a multi-parameter electronic monitor. None of the health facilities used a waveform capnography or measured the fraction of inspired oxygen, 90% never monitored Electrocardiogram (ECG) and more than 52.5% never used pulse oxymeter (Table 3). In comparison to WHO-WSFA recommendations, 28 health facilities (70%) were below standard in regard to standard monitoring and 12 health facilities (30%) had acceptable standard monitoring.

**Table 3:** Practices of monitoring during anaesthesia

Parameter	Never (%)	Sometimes (%)	Always (%)	Total (%)
BP	0 (0%)	0 (0%)	40 (100.0%)	**40 (100.0%)**
Pulse oximeter	21 (52.5%)	3 (7.5%)	16 (40.0%)	**40 (100.0%)**
ECG	36 (90.0%)	4 (10.0%)	0 (0%)	**40 (100.0%)**
Waveform Capnography	40 (100.0%)	0 (0%)	0 (0%)	**40 (100.0%)**
Fio2	40 (100.0%)	0 (0%)	0 (0%)	**40 (100.0%)**

### Causes of poor monitoring

All the participants recognized that monitoring was important during anaesthesia. The lack of equipment was the first cause stated. The causes for poor monitoring are shown in the Table 4.

**Table 4:** Causes of poor monitoring during anaesthesia

Cause of poor monitoring	ECG Never used ***N*** = 36 (%)	Pulse oximeter Never used ***N*** = 21 (%)	Complete standard Monitoring ***N*** = 40 (%)
Lack of equipment	35 (97.2)	20 (95.2)	39 (97.5)
Lack of some monitoring parts (ECG electrodes, Pulse oximeter probes, …)	18 (50.0)	11 (52.4)	21 (52.5)
Lack of information	15 (41.7)	13 (61.9)	25 (62.5)
Lack of training	21 (58.3)	18 (85.7)	22 (55.0)

## Discussion

This study documents an insufficient standard of anaesthesia monitoring during surgery in the Health Antenna of Butembo. There is no anaesthesiologist in the region and almost 50% of anaesthesia is provided by non-trained providers. About 90% of the included health facilities never used ECG and more than 50% did never use pulse oximeter. These findings are alarming and underscore the requirement for improvement and increased awareness related to safe anaesthesia and surgery. The results of this study mirror the real situation of anaesthesia monitoring in the health antenna of Butembo and can be generalized to the entire region for several reasons. In fact, the facilities in this study were among the accessible in regard of security and which probably receive most patients in the region; these health facilities were for both public and private sectors. Furthermore, the study had a high rate of answers from referral hospitals (10/17). The study also included the answer of the most senior and experienced anaesthetist who is most of the time in charge of continuing professional development in the units.

This study demonstrates that several facilities had only one operating room with the minimum number of anaesthesia providers of one. The study clearly reflects the actual situation of anaesthesia in low-income countries featured by insufficient quality of provided anaesthesia, shortage of anaesthesia providers, lack of infrastructure, drugs and equipment [3, 4, 9]. Insufficient number of providers increases the workload for the single provider available with high risk of fatigue and burnout, low standard practice and unpredictable service delivery. Moreover, the anaesthesia provider doesn’t have enough time for continuous education, professional development, administration, research, and teaching which are important and recommended by the WFSA in order to improve own practice [1, 9]. In the WFSA Global Anesthesia Workforce Survey the authors reported that the density of all anaesthesia providers in 70 countries was < 5 per 100,000, for an acceptable minimum of 5 per 100,000. DRC was part of the survey with an overall density of anaesthesia providers of 1.42 per 100,000. The rate is 1.17 per 100,000 in Malawi and 0.14 per 100,000 in Chad [10]. Moreover, there was no physician specialist practicing anaesthesia in any health facility of this study, and close to half of all the anaesthesia providers (47.5%) were nurses without any anaesthesia training, sometimes even without exposure to anaesthesia. In fact, no hospital in the Health Antenna of Butembo has a physician specialized in anaesthesia and the number of nurse anaesthetist is still low despite the presence of the faculty of anaesthesia in the ISTM. The situation is further aggravated by an increased demand in surgery and lack of qualified anaesthetists, forcing regular nurses to provide anaesthesia under supervision of the surgeons who are not specialists in anaesthesia. This is a hazardous and unsafe practice because proper monitoring requires the presence of a trained anaesthesia provider, which is paramount for the interpretation of the monitoring and safe patient care. An increased number of trained anaesthesia providers remains the key for safe anaesthesia so as to reduce perioperative morbidity and mortality [1, 3, 6–12].

As much as 57.5% (23/40) of anaesthesia providers were conducting anaesthesia alone. These results are similar to those obtain by Merry et al. [11]. Practicing alone is not a good practice. Effective teamwork is recognized as a vital component of patient safety and has demonstrated to reduce perioperative complication in high income countries [12, 13]. Efforts have to be made both by government and partners of health to address this workforce shortage, which certainly put patients at risk and may explain the high perioperative mortality rates observed in the region. Although few studies document the operative mortality in the region, a study conducted by Furaha et al. in Obstetric showed a very high mortality of 31 per 10,000 [14].

The present study demonstrates that only 30% (12/40) of health facilities were using the highly recommended standard for monitoring as suggested by WHO-WSFA. Moreover, only 40% were using a multi-parameter monitor capable of generating alarms. Although blood pressure was measured for every patient in all the facilities, a large number of the health facilities (60%) were using a manual BP machine without any alarm. No audible alarms represent a high risk for the patient safety because it may delay immediate recognition and treatment of a life threatening condition [1, 7, 11]. Furthermore, only 40 % were using a pulse oximeter during anaesthesia. This is a very low rate and reflects an unsafe practice. Anaesthesia providers have agreed that pulse oximeter should be present at all time during anaesthesia despite the fact that no robust evidence claim that this reduce perioperative mortality. Pulse oximeter may help in early detection of hypoxemia, hypovolemia and cardiac arrest and thus directing the management during anaesthesia [1, 3, 15, 16].

Almost all anaesthesia providers (97.5%) attributed their situation of poor monitoring to lack of equipment. The constraints related to equipment are still a very big challenge that needs to be addressed in order to reach safe anaesthesia and save lives in low-income countries and especially in Butembo [3, 4, 6, 9, 12]. Twenty-five anaesthesia providers (62.5%) didn’t have any information about standard monitoring. As emphasized above, nurses without any training in anaesthesia represent a non-negligible workforce in the region. They don’t have adequate skills and knowledge about anaesthesia, which is dangerous and of course represents potential harm for the patient. Thus, there is an urge to empower anaesthesia training in the region. The health stakeholders should support the local school of anaesthesia in order to address the issue of trained anaesthesia providers.

### Limitations

The large geographic health district of Butembo is partly insecure, due to presence of several rebels armed groups, and difficult to access with no practical roads making it not possible for our study to reach all health facilities in the region. This is a clear limitation to the study. However, we got more responders from the referral hospitals and big centers, which are supposed to be well equipped, and also a mix of facilities from private and public sector. Thus we have reasons to think that our findings reflect the real situation of monitoring during anaesthesia in the health facilities of the region. For intubation confirmation we relayed on auscultation and did not include capnometry in the classification of facilities. In fact, we have regarded trained providers performing intubation successfully confirmed with auscultation alone. Auscultation is part of core training in Anaesthesia and according to WHO-WFSA it is an acceptable standard to confirm endotracheal tube placement especially in limited resources countries. However, health facilities should start procuring capnographs because continuous waveform capnography has become the gold standard for intubation confirmation and is highly recommended by WHO-WFSA whenever possible. Finally, this study was limited to evaluate monitoring during anaesthesia and further research is required to assess the impact of substandard monitoring during anaesthesia on perioperative morbidity and mortality.

## Conclusions

Our study has demonstrated that there is insufficient standard of anaesthetic monitoring during surgery in the Health Antenna of Butembo. There is no anesthesiologist in the Health Antenna and anesthesia is largely practiced by non-trained providers. Most of the hospitals don’t fulfill the WFSA-WHO recommendations for standard monitoring during anaesthesia due to lack of trained personnel, equipment and knowledge. These findings reflect unsafe and high risk anaesthesia in the region. This is alarming and underscore the requirement for improvement and increased awareness related to safe anaesthesia and surgery in the region. This can be achieved by enhanced training of more anaesthesia providers, both physicians and non-physicians, and a substantial investment in anaesthesia equipment.

## Supplementary information

**Additional file 1.** Questionnaire Anaesthesia Monitoring Butembo North Kivu, Data collection form.

## Data Availability

All data and material used in this article are available at any request from the corresponding author.

## Abbreviations

BP: Blood pressure
DRC: Democratic Republic of Congo
ECG: Electrocardiogram
ISTM: Institut Superieur des techniques médicales
LMD: License Master Doctorate
min: Minimum
max: Maximum
WFSA: World Federation of Societies of Anaesthesiologists
WHO: World Health Organization
