# Supplementary material for: Practice of standard monitoring during anaesthesia in hospitals of North Kivu: a survey of health facilities of the health antenna of Butembo
Source: BMC Health Serv Res. 2020 Mar 30;20:262. doi: 10.1186/s12913-020-05076-x (PMC7106833; doi:10.1186/s12913-020-05076-x)
Supplement: Supplementary file 1 — Additional file 1. Questionnaire Anaesthesia Monitoring Butembo North Kivu, Data collection form. [file 12913_2020_5076_MOESM1_ESM.docx]

**DATA COLLECTION FORM**

**“Practice of standard monitoring during anaesthesia in hospitals of North Kivu”**

We are seeking for your participation in this study which is assessing the practice of standard monitoring during anaesthesia in your area in order to improve the practice of monitoring and safety during anaesthesia. This study received approval from the “Comité Ethique du Nord Kivu” as well as from the Health Antenna of Butembo. This is a free of charge and anonymous study. There is no risk to participate in this study and your confidentiality will be protected anytime during data processing and report.

0. Would you like to participate? a. Yes b. No **If No Go to question 20.0**

1. Health Zone: …………………...

2. Health Facility: ………………………… 3. Sector a. Public b. Private/Faith based

4. Would you please tell us your Age: ……. years 5. Gender a. Male b. Female

6.What is your level of training:

a. Graduate b. “Licencié” c. Nurse Practicing anaesthesia d. Doctor(Dr.) e. Other……………

7. For how long have you been practicing anaesthesia? …….. months/years **(Underline the unit)**

8. How many operating rooms do you have in your facility? ………..

9. How many providers are practicing anaesthesia in your facility?

a. Number of Nurses Anaesthetists: ………….

b. Number of Doctors Anaesthetists: ……….

c. Number of other Nurses practicing Anaesthesia: ………...

d. Number of other Doctors practicing anaesthesia: ………...

10. Do you provide general anaesthesia in your facility? a. Yes b. No

11. Do you provide spinal anaesthesia in your facility? a. Yes b. No

12. How many anesthetists are there present during anaesthesia?

a. Only one b. One with help of a nurse c. Nurse under Dr. supervision d. sometimes 2 e. Always 2 or more

13. Do you have a stethoscope in your operating room all the time? a. Yes b. No

14. Which kind of monitor are you using?

a. Electronic multi-parameter monitor

b. Each parameter with own monitor

15. How many operating rooms have the electronic multi-parameter monitor? .....

16. If 13 answer is b, for blood pressure (BP) measurement, which BP machine are using? a. Manual b. Electronic

17. Which parameter are you routinely monitoring during general anaesthesia? **(Tick off)**

| **Parameter** | **Never** | **Sometimes** | **Always** |
| --- | --- | --- | --- |
| ECG |  |  |  |
| Blood pressure |  |  |  |
| Oxygen Saturation |  |  |  |
| Temperature |  |  |  |
| Waveform Capnography |  |  |  |
| Fraction of inspired oxygen |  |  |  |

18. Which parameter are you routinely monitoring during spinal anaesthesia? **(Tick off)**

| **Parameter** | **Never** | **Sometimes** | **Always** |
| --- | --- | --- | --- |
| ECG |  |  |  |
| Blood pressure |  |  |  |
| Oxygen Saturation |  |  |  |
| Temperature |  |  |  |

19. What are the reasons why you don’t monitor all these parameters? **(Tick off one or more)**

| **Reason** | **ECG** | **BP** | **Pulse oximetry** | **Complete monitoring** |
| --- | --- | --- | --- | --- |
| Not necessary |  |  |  |  |
| lack of material |  |  |  |  |
| lack of some components |  |  |  |  |
| lack of electricity |  |  |  |  |
| lack of information |  |  |  |  |
| Lack of training |  |  |  |  |
| Other to specify |  |  |  |  |

**20.0. Thank you very much for your time.** If you have any question or preoccupation, please feel free to contact the research team on these numbers: **+243 999 146 806, +243 993 078 764.**
